# Associations between hepcidin and immune response in individuals with hyperbilirubinaemia and severe malaria due to *Plasmodium vivax* infection

**DOI:** 10.1186/s12936-015-0930-x

**Published:** 2015-10-14

**Authors:** Vitor R. R. Mendonça, Ligia C. L. Souza, Gabriela C. Garcia, Belisa M. L. Magalhães, Marilda S. Gonçalves, Marcus V. G. Lacerda, Manoel Barral-Netto

**Affiliations:** Laboratório Integrado de Microbiogia e Imunoregulação (LIMI), Centro de Pesquisas Gonçalo Moniz, Fundação Oswaldo Cruz (FIOCRUZ), Salvador, Brazil; Faculdade de Medicina, Universidade Federal da Bahia, Salvador, Brazil; Fundação de Medicina Tropical Dr Heitor Vieira Dourado, Manaus, Brazil; Universidade do Estado do Amazonas, Manaus, Brazil; Instituto de Investigação em Imunologia, Instituto Nacional de Ciência e Tecnologia, São Paulo, Brazil

**Keywords:** Malaria, *Plasmodium vivax*, Immune response, Hepcidin, Hyperbilirubinemia

## Abstract

**Background:**

Hyperbilirubinaemia (bilirubin >51.3 μmol/L) alone is not indicative of severe malaria, and the immune response underlying hyperbilirubinaemia remains largely unexplored. Liver damage associated with hyperbilirubinaemia may alter the expression of hepcidin, which regulates systemic iron by degrading ferroportin. For this study, the association between hepcidin and the levels of cytokines and chemokines in the serum of individuals with mild and severe vivax malaria and subjects with malaria with isolated hyperbilirubinaemia was evaluated.

**Methods:**

Cytokines/chemokines and hepcidin were measured in individuals with mild (n = 72) and severe (n = 17) vivax malaria, as well as in the serum of subjects with vivax malaria with isolated hyperbilirubinaemia (n = 14) from the Brazilian Amazon between 2009 and 2013 by multiplex assay and ELISA, respectively. The polymorphism 744 G > T in the *ferroportin* gene was identified by restriction fragment-length polymorphism analysis and the restriction enzyme *Pvu*II.

**Results:**

The polymorphism at position 744 G > T in the *ferroportin* gene was typed and no differences in the distributions of genotypes or alleles were observed between the study groups. Subjects with severe malaria had higher levels of interleukin (IL)-2 and IL-13 than subjects with hyperbilirubinaemia. No differences in the expression of immune markers were observed between subjects with mild malaria and those with hyperbilirubinaemia. However, hepcidin levels were higher in individuals with severe malaria and hyperbilirubinaemia than those with mild malaria (p = 0.0002 and p = 0.0004, respectively) and cut-off values of hepcidin differentiated these groups from subjects with mild malaria. Hepcidin was positively associated with IL-6 and IL-10 levels and with parasitaemia in subjects with mild malaria and with IFN-γ in subjects with severe malaria.

**Conclusions:**

Malaria in the presence of hyperbilirubinaemia produces a less robust inflammatory response compared to severe cases of malaria. Hepcidin levels are positively associated with immune markers in vivax malaria outcomes.

**Electronic supplementary material:**

The online version of this article (doi:10.1186/s12936-015-0930-x) contains supplementary material, which is available to authorized users.

## Background

Malaria is a major health problem worldwide, and in 2013 it was estimated to cause 584,000 deaths [1]. *Plasmodium vivax* is the most widespread malaria parasite and is responsible for the majority of malaria cases in Southeast Asia and South America [1]. Clinical outcomes from *Plasmodium* infections can range from severe or mild diseases to asymptomatic parasite carriers. The balance and interactions between anti- and pro-inflammatory cytokines play an important role in vivax malaria manifestations [2]. Further, genetic alterations in genes related to immune response have been associated with clinical outcomes [3, 4].

Hyperbilirubinaemia, which is also known as jaundice, is often associated with malaria infection. It occurs as a consequence of the intravascular haemolysis, disseminated intravascular coagulation and hepatocellular dysfunction [5]. However, recently, jaundice (serum total bilirubin >3 mg/dL) is no longer considered a single marker of malaria severity and the presence of hyperbilirubinaemia alone is not associated with a worse prognosis or a higher fatality rate in malaria [5–8]. Nevertheless, hyperbilirubinaemia is a common complication associated with severe malaria syndromes and concomitant jaundice can indicate more severe illness [9–12].

The rupture of red blood cells in the blood stream during malaria infection is associated with an increase in indirect bilirubin, but the primary schizogony of the malarial parasite also leads to the rupture of infected hepatocytes and elevates direct bilirubin levels. Both of these factors contribute to hyperbilirubinaemia and clinical jaundice [12–16]. In this context, the hepatocellular damage observed in individuals with malaria and hyperbilirubinaemia may alter the hepatocytic expression of hepcidin, which regulates systemic iron homeostasis by degrading ferroportin, the only known human iron cell exporter [17]. The degradation of ferroportin leads to the inhibition of intestinal absorption of dietary iron and accumulation of iron in macrophages leading to low iron availability [17].

The immune response underlying malaria-related jaundice, which is defined by high productions of interleukin (IL)-6, IL-10 and interferon (IFN)-γ, may influence hepcidin levels in hepatocytes and peripheral blood mononuclear cells [18, 19]. Portugal et al. described that increased hepcidin levels during a *Plasmodium* blood-stage infection inhibited subsequent liver infection in a rodent model [20]. Further, hepcidin levels are regulated by inflammation, hypoxia, iron status, and IL-6 production [21–27]. Hepcidin levels are increased in children during acute *P. falciparum* uncomplicated malaria [23] as well asymptomatic malaria caused by *P. falciparum* or *P. vivax* [24]; however, children with severe falciparum malaria demonstrated to have very low levels of this hormone [25–27]. Recently, hepcidin levels were demonstrated to be the best predictor of iron absorption in children under competing conditions, such as anaemia, iron deficiency and infection [28]. This suggests a potential utility for hepcidin in managing iron supplementation programmes at the time of malaria infection because of the inhibitory effect of hepcidin on the absorption of oral iron.

Noteworthy, no previous studies involving hepcidin in adults with symptomatic vivax malaria and in adults with severe malaria have been done so far. Hepcidin has been found to be low in children with severe malaria [25–27], but adults with severe malaria have not been evaluated. In the present study, it was studied the associations between hepcidin and the levels of cytokines and chemokines in the serum of adults with severe and mild vivax malaria, as well as in subjects with vivax malaria with isolated hyperbilirubinaemia. The results herein indicate that individuals with hyperbilirubinaemia alone exhibit an inflammatory response similar to subjects with mild infections without hyperbilirubinaemia. Of equal importance is the finding that subjects with malaria and hyperbilirubinaemia and subjects with severe malaria have elevated levels of hepcidin; cut-off values of hepcidin can differentiate these groups of subjects from those with mild malaria. Further, hepcidin levels are associated with immune responses in the different *P. vivax* infection study groups.

## Methods

### Study design and participants

Individuals with an acute febrile syndrome (age range, 4 months to 76 years; median age 36 years) who sought care at the reference hospital at the Fundação de Medicina Tropical Dr Heitor Vieira Dourado (FMT-HVD), Amazonas, Brazil, were recruited between 2009 and 2013. Subjects were tested for malaria by thick blood smear and those with *P. vivax* infections confirmed by PCR were invited to participate in this study. All patients with vivax malaria who developed haemolysis due to primaquine treatment (i.e., patients using primaquine who experienced haemoglobin <10 g/dL and reticulocyte count >1.5 % or an indirect increase in bilirubin levels after using primaquine) were excluded. Patients with microscopic or molecular diagnosis of malaria caused by *Plasmodium falciparum* or *P. vivax* and *P. falciparum* co-infection and patients with a serologic diagnosis of viral hepatitis (A, B, C, or D), HIV, or leptospirosis were also excluded. Participants were categorized into three groups based on the severity of infection: mild malaria (n = 72; without hyperbilirubinaemia), malaria with hyperbilirubinaemia (n = 14) and severe malaria (n = 17). Severe vivax malaria was defined according to the criteria for severe malaria established by the World Health Organization (WHO); malaria with hyperbilirubinaemia was defined by a serum total bilirubin level >51.3 μmol/L with no other criteria for severe malaria [6]. Details of subjects with severe malaria and hyperbilirubinaemia can be found in Mendonça et al. [3].

### Ethics statement

This study was approved by the Ethics Committee of the FMT-HVD (protocol number: 2009/15243) and all subjects provided written informed consent. All clinical investigations were conducted according to the principles outlined in the Declaration of Helsinki.

### Plasma measurements

Blood was obtained by venipuncture; heparinized plasma was separated and immediately used or stored at −70 °C. The following clinical markers were measured in fresh plasma samples at the clinical laboratory facility at the FMT-HVD (Manaus, Brazil): haemoglobin (HB), haematocrit (HT), platelets (PTL), aspartate aminotransferase (AST), and alanine aminotransferase (ALT). Levels of hepcidin in thawed plasma were measured by enzyme-linked immunoassay (Assay Designs, Ann Arbor, MI, USA). Plasma levels of IL-1β, IL-2, IL-4, IL-5, IL-6, IL-7, IL-8, IL-10, IL-12p70, IL-13, IL-17, IFN-γ, tumor necrosis factor (TNF), chemokines CCL2 and CCL4, granulocyte-colony stimulating factor (GCSF), and granulocyte–macrophage colony-stimulating factor (GMCSF) were measured using a multiplex assay according to the manufacturer’s protocol (Bio-Rad, Hercules, CA, USA).

### Genotyping

DNA was extracted from 200 μL of peripheral blood using a standard QIAGEN DNA blood mini kit (QIAGEN, Valencia, CA, USA) according to the manufacturer’s protocol. The polymorphism at position 744 G > T (rs11568350; G ancestral allele) in the *ferroportin* gene (*SLC40A1*) was typed by polymerase chain reaction-restriction fragment length polymorphism (PCR–RFLP) analysis with the restriction enzyme *Pvu*II (New England Biolabs, Ipswich, MA, USA) according to the protocol previously published by Kasvosve et al. [29]. *SLC40A1* PCR products were size-separated by electrophoresis on 3 % agarose gel under non-denaturing conditions. PCR products were subsequently stained with ethidium bromide and visualized under a UV light.

### Network analyses

Networks were generated from Spearman correlation matrices that contained values of each plasma marker measured in the samples; values were input into JMP 10.0 software (SAS, Cary, NC, USA). Each marker was selected as a target and the software performed a search within the other mediators for those that were correlated with the target and calculated a correlation matrix using Spearman rank tests. The features related to the selected target were linked, and the links shown in the networks represented statistically significant Spearman rank correlations (P < 0.05). To analyse the structure of the marker’s network, the density of each network was calculated (range, 0–1). In the context of this study, the density was the ratio of the number of edges inferred in the network over the total number of possible edges between all pairs of nodes [2]. The network’s figures were customized using the Ingenuity Systems Pathway Analysis software (Ingenuity Systems, Redwood City, CA, USA) and Adobe Illustrator (Adobe Systems Inc.).

### Statistical analyses

Chi square or Fisher exact tests were applied to evaluate the associations between qualitative variables. The D’Agostino-Pearson omnibus normality test was used to test for Gaussian distribution of quantitative variables within the total sample. Variables that were not normally distributed were analysed with non-parametric tests. The Kruskal–Wallis test with Dunn’s multiple comparison (when three groups were compared) and the Mann–Whitney test (when two groups were compared) were used to assess the differences among the clinical groups. Multinomial regression analyses adjusted for age and gender were performed to test associations between the plasma measurements (below or above the median values of the entire study population) and the different clinical conditions (mild malaria, hyperbilirubinaemia and severe malaria). The receiver operating characteristic (ROC) curves and C-statistics of markers were used to test the ability to distinguish between the different clinical groups. A hierarchical cluster analysis using Ward’s method was performed to test whether a combination of different immune-related biomarkers could cluster the study groups separately. Hardy–Weinberg equilibrium (HWE) was assessed for the groups by comparing the observed number of different *SLC40A1* genotypes with those expected under HWE for the estimated allele frequency. Statistical analyses were performed using GraphPad Prism 6.0 (GraphPad Software Inc., USA), SPSS 19.0 (IBM, Armonk, NY, USA), and JMP 11.0 (SAS, Cary, NC, USA). A *p* value <0.05 was considered statically significant.

## Results

### Baseline characteristics and *SLC40A1* 744 G > T distribution of the study participants

Most of the study subjects were male: 77.78 % (n = 56) of the mild malaria group, 71.43 % (n = 10) of the malaria with hyperbilirubinaemia group and 52.94 % (n = 9) of the severe malaria group (p = 0.0592; Table 1). There were no significant differences in age among the study groups (p = 0.1274; Table 1). Parasitaemia levels were similar among the groups (p = 0.2115; Table 1). Subjects with severe malaria displayed lower levels of HB and HT than subjects with hyperbilirubinaemia and subjects with mild malaria (p < 0.0001 for both comparisons; Table 1). Individuals with hyperbilirubinaemia had the lowest PTL values (p = 0.0003) and the highest ALT values (p = 0.0039); AST levels did not differ among the groups (p = 0.0686; Table 1).

**Table 1:** Baseline characteristics, laboratory measurements, and distribution of *SLC40A1* 744 (G > T) polymorphism in the study participants

	Mild malaria	Malaria with hyperbilirubinaemia	Severe malaria	P-value
	(n = 72)	(n = 14)	(n = 17)	
Male, n (%)	56 (77.78)	10 (71.43)	9 (52.94)	0.0592**
Age (year), median (IQR)	36.00 (27.00–45.50)	26.00 (20.25–36.25)	37.00 (10.50–44.50)	0.1274***
Parasitaemia (parasites/μL), median (IQR)	2673 (842–9313)	2,451 (247–11,655)	291 (0–29,184)	0.2115***
Laboratory measurements, median (IQR)				
Haemoglobin (g/dL)	13.20 (12.50–14.28)	11.75 (10.38–13.88)	7.10 (6.50–10.50)	<0.0001***
Haematocrit (%)	43.50 (40.65–46.18)	34.10 (30.20–41.45)	23.10 (19.75–31.90)	<0.0001***
Platelets (per mm3)	108,500 (71,250–132,250)	33,000 (18,000–51,250)	64,000 (33,500–202,000)	0.0003***
AST (IU/L)	67.50 (50.00**–**93.25)	65.00 (36.00–172.50)	34.00 (21.50–112.30)	0.0686***
ALT (IU/L)	34.00 (19.25–50.75)	104.50 (36.00–262.50)	41.50 (13.25–76.00)	0.0039***
*SLC40A1* 744 (G > T) n (%)				
GG	71 (98.61)	14 (100.00)	16 (94.12)	0.4108**
GT	1 (1.39)	0 (0.00)	1 (5.88)	
TT	0 (0.00)	0 (0.00)	0 (0.00)	
G allele	141 (99.30)	28 (100.00)	33 (97.06)	0.4199**
T allele	1 (0.70)	0 (0.00)	1 (2.94)	

*ALT* alanine aminotransferase, *AST* aspartate aminotransferase, *IQR* interquartile range

** Categorized variables were compared using the Chi square test

*** Ordinal variables were compared using the Mann–Whitney test for two groups or the Kruskal–Wallis test with Dunn’s multiple comparison for three or more groups

A very low frequency of the *ferroportin*-associated polymorphism (*SLC40A1* 744 G > T) was identified in the study subjects (Table 1). No individuals with the homozygous mutant-type (TT) genotype were identified; one (1.39 %) heterozygous individual (GT genotype) was identified in the mild malaria group and one (5.88 %) was identified in the severe malaria group (p = 0.4108; Table 1). No differences were observed in allele distributions among the study groups (p = 0.4199; Table 1). The frequencies of *SLC40A1* 744 G > T genotypes met the conditions for HWE in all categories of vivax malaria infection.

### The use of hepcidin levels to differentiate subjects with severe malaria and hyperbilirubinaemia from subjects with mild malaria

**Fig. 1:**
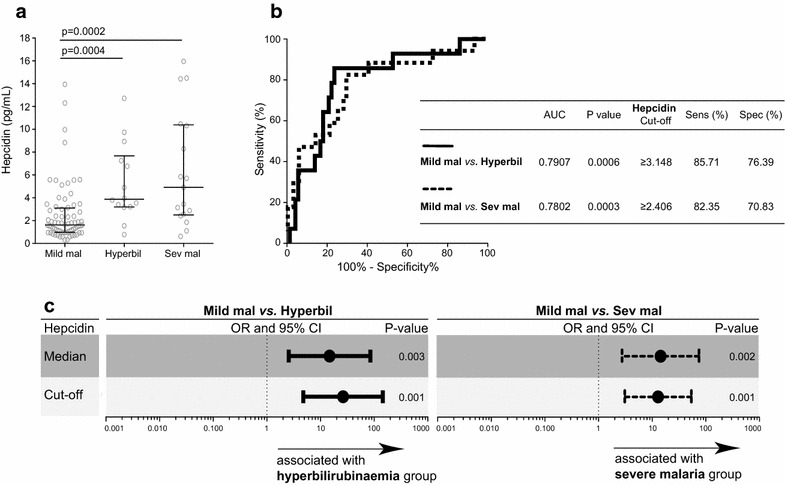
Hepcidin levels discriminate severe malaria and hyperbilirubinaemia from mild malaria. Hepcidin levels were compared in the study groups by the Mann–Whitney test (**a**). Hepcidin levels were analysed by receiver operating characteristic (ROC) curves to establish the area under the curve (AUC) and the cut-off sensitivity and specificity values to distinguish between individuals with severe malaria or hyperbilirubinaemia and individuals with mild malaria (**b**). Based on hepcidin cut-off (obtained in **b**) and median values, a multinomial regression analysis adjusted for age and gender was conducted to calculate odds ratios (ORs) and 95 % confidence intervals (CI), represented by the *icons* and *bars*, respectively (**c**). A p-value <0.05 was considered statistically significant. *Mild mal* mild malaria group, *hyperbil* hyperbilirubinaemia group, *sev mal* severe malaria group

Plasma levels of hepcidin were higher in individuals with severe malaria and hyperbilirubinaemia than subjects with mild malaria (p = 0.0002 and p = 0.0004, respectively; Fig. 1a). No difference in hepcidin levels was observed between the hyperbilirubinaemia and severe malaria groups (p = 0.9093). The ROC curves analysis and the C-statistics allowed for the calculation of the discriminatory power of hepcidin to distinguish subjects with hyperbilirubinaemia from those with severe malaria or mild malaria (Fig. 1b). Hepcidin levels >3.148 ng/mL offered a sensitivity of 85.71 %, a specificity of 76.39 % and an area under the curve (AUC) of 79.07 % to differentiate mild from severe malaria (Fig. 1b). Hepcidin levels >2.406 ng/mL offered a sensitivity of 82.35 %, a specificity of 70.83 % and an AUC of 78.02 % to differentiate mild malaria from malaria with hyperbilirubinaemia (Fig. 1b). Hepcidin median and cut-off values were established (Fig. 1b); an age- and gender-adjusted multinomial logistic regression analysis was conducted on the basis of these values to confirm the associations between high hepcidin levels and susceptibility to hyperbilirubinaemia and severe malaria (Fig. 1c). Hepcidin levels above the cut-off (3.148 ng/mL; OR 26.405, 95 % CI 4.789–145.596 ng/mL, p = 0.001) or median (1.810 ng/mL; OR 14.771, 95 % CI 2.563–85.120 ng/mL, p = 0.003) values were associated with hyperbilirubinaemia more frequently than mild malaria (Fig. 1c). Likewise, hepcidin levels above the cut-off (2.406 ng/mL; OR 12.861, 95 % CI 3.082–53.668 ng/mL, p = 0.001) or median (1.842 ng/mL; OR 14.332, 95 % CI 2.754–74.582 ng/mL, p = 0.002) values were associated with severe malaria more frequently than with mild malaria (Fig. 1c). Hepcidin concentrations of subjects in the smaller groups within the severe malaria group were not analysed due to the low number of individuals and the low power.

### The expression of immune-related biomarkers in each of the study groups

**Fig. 2:**
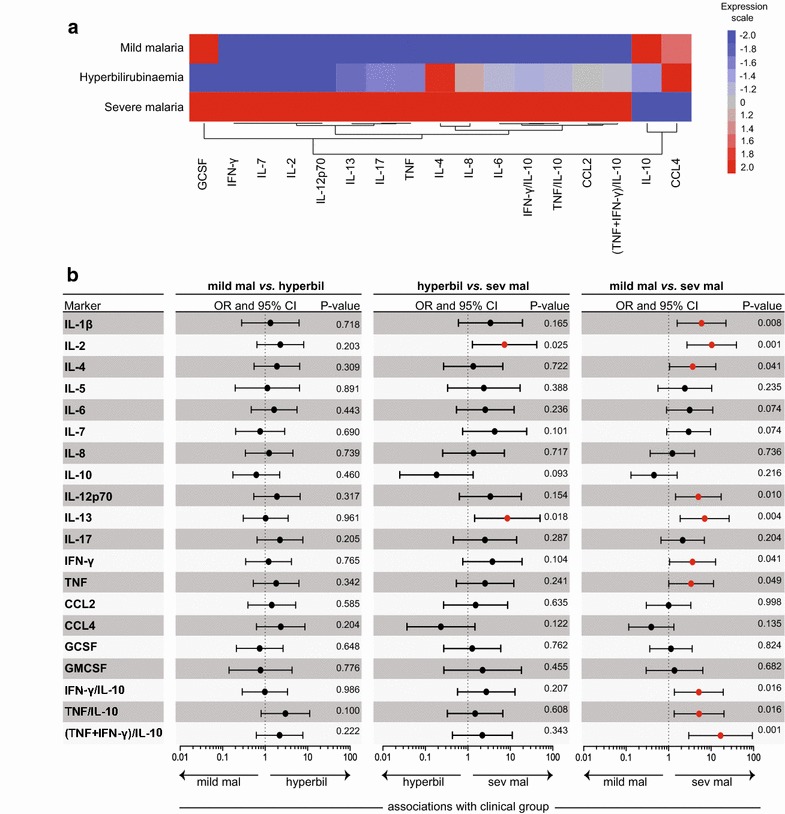
Concentration of cytokines and chemokines according to study groups. A heat map was designed to depict the overall pattern of expression of immune markers in the different study groups according to the median value of each parameter (**a**). A two-way hierarchical cluster analysis (Ward’s method) of immune molecules by clinical group was performed (**a**). Biomarkers that had the same median in the three groups were excluded from the heat map and cluster analysis. The *colours* shown for each symbol represent the fold variation from the median values calculated for each marker (**a**). Differentiation between mild malaria and hyperbilirubinaemia, hyperbilirubinaemia and severe malaria, and mild malaria and severe malaria were noted by cytokine and chemokine levels using a multinominal regression analysis adjusted for age and gender that calculated odds ratios (ORs) and 95 % confidence intervals (CI), represented by the *icons* and *bars*, respectively (**b**). *Red icons* represent a statistically significant difference. A p-value <0.05 was considered statistically significant

A panel of 17 cytokines and chemokines (IL-1β, IL-2, IL-4, IL-5, IL-6, IL-7, IL-8, IL-10, IL-12p70, IL-13, IL-17, IFN-γ, TNF, CCL2, CCL4, GCSF, GMCSF) and inflammatory ratios (TNF/IL-10, IFN-γ/IL-10 and (TNF + IFN-γ)/IL-10) were used to build a heat map to identify unique signatures that could highlight differences between the study groups in a hierarchical cluster analysis (Fig. 2a). Among the clinical groups evaluated, individuals with severe malaria exhibited the highest median concentrations of the majority of the immune markers (GCSF, IFN-γ, IL-7, IL-2, IL-12p70, IL-13, IL-17, TNF, IL-4, IL-8, IL-6, IFN-γ/IL-10, TNF/IL-10, CCL2, and (TNF + IFN-γ)/IL-10; Fig. 2a). Subjects with mild malaria exhibited the highest plasma levels of IL-10 (Fig. 2a).

Multinominal regression analyses adjusted for age and gender revealed that severe malaria was associated with higher levels of IL-2 (p = 0.025) and IL-13 (p = 0.018) than malaria with hyperbilirubinaemia (Fig. 2b). Severe malaria was also associated with higher levels of IL-1β (p = 0.008), IL-2 (p = 0.001), IL-4 (p = 0.041), IL-12p70 (p = 0.010), IL-13 (p = 0.004), IFN-γ (p = 0.041), TNF (p = 0.049), IFN-γ/IL-10 (p = 0.016), TNF/IL-10 (p = 0.016) and (TNF + IFN-γ)/IL-10 (p = 0.001) than mild malaria (Fig. 2b). No differences in markers of immune expression were observed between the mild malaria and the hyperbilirubinaemia groups according to multinomial regression analyses (Fig. 2b).

### A network analysis of immune response

**Fig. 3:**
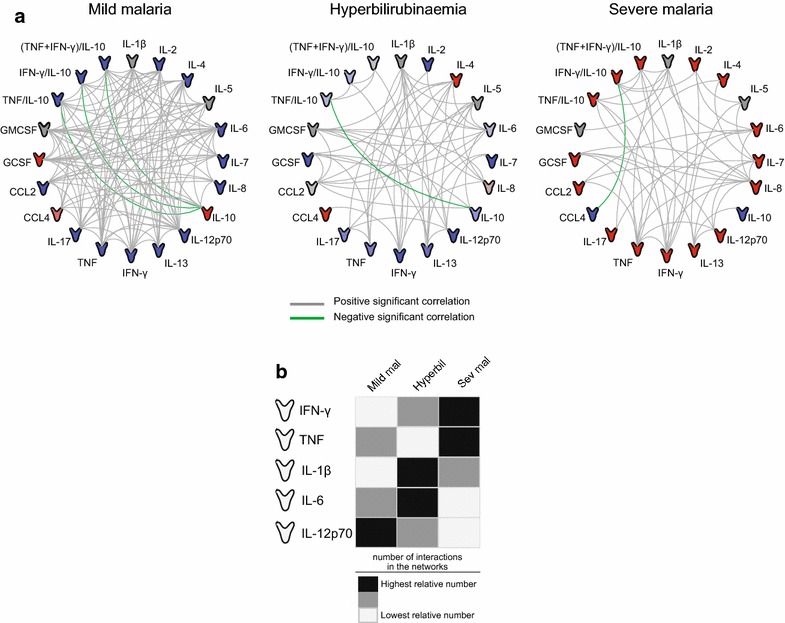
Networks of immune-related biomarkers in mild malaria, hyperbilirubinaemia and severe malaria groups. Plasma levels of several immune-related (cytokines and chemokines) biomarkers were measured in subjects with mild malaria, hyperbilirubinaemia, and severe malaria. Each connecting line represents a significant interaction (p < 0.05) detected by Spearman’s correlation test (**a**). *Grey lines* represent positive significant interactions and *green lines* represent negative significant correlations. *Icons* are colored according to their expression in the heat map shown in Fig. 2a. The five immune-related biomarkers with the highest number of interactions in all three groups were chosen (IFN-γ, TNF, IL-1β, IL-6, and IL-12p70) and the relative number of interactions of these biomarkers was calculated for each group (**b**). *Dark grey rectangles* represent the highest relative number of connections, *light dark rectangles* represent the medium relative number of connections, and *white rectangles* represent the lowest relative number of connections between molecules (**b**)

Cytokine and chemokine levels were used to build networks representing possible interactions between the candidate biomarkers in each study group. The distributions of plasma concentrations of the cytokines and chemokines in each of the different clinical groups are provided (see Additional file 1). The network analysis revealed five negative correlations between candidate biomarkers in the study groups; four of these interactions were between IL-10 and its inflammatory ratios (IL-10 as a dividend). A majority of the statistically significant correlations observed were positive (Fig. 3a). However, the densities of the networks from each clinical group were different. The mild malaria group exhibited the highest density of interactions (network density: 0.605), which may be due to the higher number of individuals in this group compared to the other groups. The hyperbilirubinaemia group exhibited a network density of 0.284 and the severe malaria group exhibited the lowest density of 0.253. P-values and Spearman rank values for each correlation of immune biomarkers according to study groups are detailed (see Additional file 2).

Of the 20 immune-related biomarkers and inflammatory ratios assessed in the network analyses, five cytokines (IFN-γ, TNF, IL-1β, IL-6, and IL-12p70) exhibited the most interactions (statistically significant Spearman correlations) across all groups. IFN-γ participated in 13.82 % of all interactions, TNF in 13.36 % of all interactions, IL-1β in 15.21 % of all interactions, IL-6 in 13.82 % of all interactions, and IL-12p70 in 12.44 % of all interactions (Fig. 3b). In order to assess if the number of network connections involving each of these cytokines could highlight differences among the clinical groups, the percentage of edges involving each molecule as a portion of the overall number of edges in the network was calculated. IFN-γ and TNF showed the highest relative number of network interactions in the severe malaria group and IL-1β and IL-6 showed the highest relative number of interactions in the hyperbilirubinaemia group (Fig. 3b). IL-12p70 showed the highest number of interactions in the mild malaria group (Fig. 3c). These results suggest that unique immune signatures involving plasma cytokine and chemokine levels highlight differences that differentiate mild malaria, malaria with hyperbilirubinaemia, and severe malaria.

**Fig. 4:**
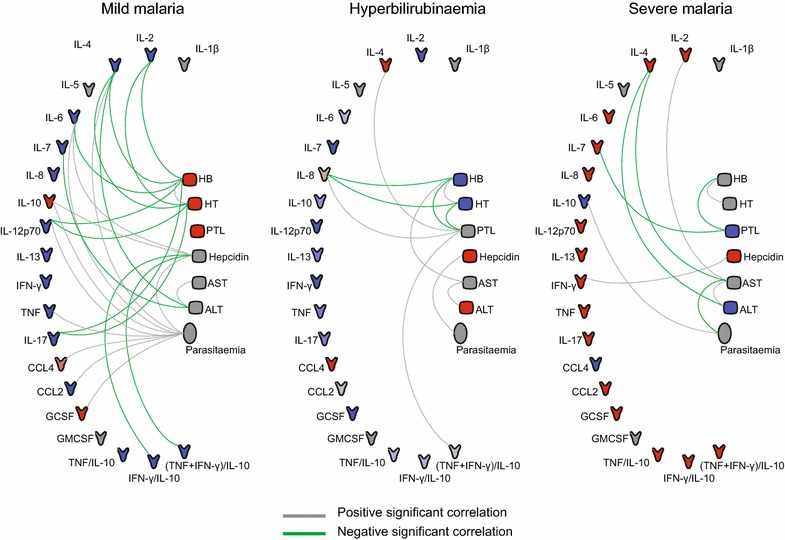
Associations between laboratory parameters, parasitaemia, hepcidin levels, and immune-related biomarkers. Statistically significant correlations between laboratory markers, parasitaemia, hepcidin levels, and immune-related biomarkers are shown for each study group. The correlations were assessed using the Spearman rank test. *Green lines* represent negative significant (p < 0.05) correlations and *grey lines* represent positive significant correlations. Icons for cytokines and chemokines are *coloured* according to their expression in the heat map shown in Fig. 2a. The other icons are *coloured* according to their expressions in the comparisons between study groups: *grey* icons are parameters with no difference between the two groups; *blue* and *red* icons represent downregulated and upregulated markers, respectively

Next, the interactions between clinical laboratory markers, parasitaemia, hepcidin levels, and immune-related molecules were evaluated (Fig. 4). As expected, in all study groups, HB levels were positively correlated with HT and ALT was positively correlated with AST (Fig. 4). Hepcidin was positively correlated with IL-6 (r = 0.4339; p = 0.0015), IL-10 (r = 0.5154; p = 0.0002), and parasitaemia (r = 0.3032; p = 0.0096) and negatively correlated with IFN-γ/IL-10 (r = −0.4369; p = 0.0017) and (TNF + IFN-γ)/IL-10 (r = −0.3776; p = 0.0075) in the mild malaria group. Hepcidin was positively correlated with parasitaemia (r = 0.5912; p = 0.0260) in the hyperbilirubinaemia group and with IFN-γ (r = 0.5324; p = 0.0278) in the severe malaria group. P-values and Spearman rank values for each correlation between immune biomarkers, laboratory measures, parasitaemia, and hepcidin levels are provided (see Additional file 3).

## Discussion

Relationships between plasma markers of immune response and hepcidin levels during severe vivax malaria and malaria with hyperbilirubinaemia are largely unexplored. The results of the present study indicate that plasma hepcidin levels are higher in individuals with severe vivax malaria and individuals with hyperbilirubinaemia than in those with mild malaria; cut-off values of hepcidin can discriminate malaria outcomes. However, individuals with malaria with hyperbilirubinaemia had plasma cytokine and chemokine levels similar to those of individuals with mild malaria. Subjects with hyperbilirubinaemia exhibited a less robust inflammatory response than subjects with severe malaria, which reinforces the exclusion and adaptation of this condition in the most recent WHO malaria severity criteria [6]. Hepcidin was positively associated with IL-6, IL-10, and parasitaemia in the mild malaria group and with IFN-γ in the severe malaria group, which suggests a possible role of immunological regulation in the expression of this hormone.

Jaundice is a clinical outcome often associated with malaria infection, but the exact pathogenesis of this condition is not clearly understood [5]. The rupture of red blood cells, ischemia due to adherence of parasitized erythrocytes to the endothelial walls, and, to a lesser extent, hepatocellular dysfunction can contribute to hyperbilirubinaemia [9]. The present study confirms that individuals with hyperbilirubinaemia in the context of vivax malaria have low levels of PTL and high levels of ALT, which suggests that a degree of liver dysfunction associated with this condition might influence hepcidin expression by hepatocytes [12–16]. Hyperbilirubinaemia is not itself a complication of severe malaria, but it is associated with higher morbidity and mortality when it occurs concomitantly with at least one other complication [5, 8, 11]. In this study, individuals infected by *P. vivax* with hyperbilirubinaemia had lower levels of IL-2 and IL-13 than individuals with severe cases of malaria; no differences in immune response were observed between subjects with hyperbilirubinaemia and those with mild malaria. Even though the hyperbilirubinaemia group demonstrated an overall low immunoinflammatory status, the immune response may still have an important role in the pathogenesis of hyperbilirubinaemia. In the network analysis, IL-1β and IL-6 were highly associated with other inflammatory parameters. In fact, these markers may regulate the quality of the immune response and the high hepcidin levels associated with hyperbilirubinaemia. Levels of IL-6 have already been associated with severe malaria outcomes, and jaundice has been demonstrated to be independently linked to increased levels of this cytokine [18, 30]. Furthermore, IL -6 appears to be correlated with hepcidin levels during malaria infection [22, 25–27].

Traditionally, malaria by *P. vivax* has been considered a benign infection, but, in recent years, *P. vivax* malaria has been increasingly associated with severe disease [31–33]. The immune response, with its balance of anti- and pro-inflammatory mediators, seems to play an essential role in severe vivax malaria [2]. As expected, in the present study, individuals with severe vivax malaria exhibited higher concentrations of immune markers. This group also demonstrated a significant interaction between IFN-γ and TNF. Elevated levels of TNF and IFN-γ are related to severe forms of infection, such as cerebral malaria and severe anaemia in *P. falciparum* malaria [18, 33, 34]. In contrast, subjects with mild malaria displayed herein higher levels of IL-10 than subjects with severe malaria. These findings confirm previous studies that indicated a role of this regulatory cytokine in mild malaria outcomes [33, 35].

Hepcidin is the major regulator of iron levels, which it accomplishes by degrading the ferroportin receptor and inhibiting iron release from intracellular compartments. Mutations in the *ferroportin* gene (*SLC40A1*) have been linked to high iron stores and haemochromatosis in humans influencing hepcidin levels [29, 36, 37]. In this study population, the *SLC40A1* SNP (744 G > T) was rarely detected and it did not influence vivax malaria outcomes. Several studies have shown that hepcidin levels are increased in asymptomatic and symptomatic malaria caused by *P. falciparum* or *P. vivax* [21–24, 38]. In this study, higher plasma levels of hepcidin were observed in subjects with severe malaria and malaria with hyperbilirubinaemia than in subjects with mild malaria. The increased hepcidin levels in the malaria with hyperbilirubinaemia group may be a consequence of liver damage, hepatocellular dysfunction, and the immune response associated with hyperbilirubinaemia. Despite the possibility of liver dysfunction influencing hepcidin levels [18, 19] in malaria with hyperbilirubinaemia group, it was not observed a direct correlation between hepcidin and ALT or AST levels, and other liver biomarkers cannot be explored due to limitation of the volume of stored plasma.

Recently, hepcidin was discovered to be the best predictor of erythrocyte iron incorporation, which suggests a role for this hormone in the management of iron supplementation programmes [28]. Hepcidin cut-off values may also be used to distinguish severe malaria from mild malaria (without hyperbilirubinaemia) outcomes in endemic settings, as demonstrated in this study. Further, hepcidin cut-off values can be an additional tool (biomarker) in addition to the WHO criteria to distinguish severe or hyperbilirubinaemia patients from mild infection. Interestingly, some studies have reported that individuals with severe falciparum malaria, defined by severe anaemia and cerebral malaria, have low hepcidin levels [25–27]. Such differences in hepcidin concentrations may be explained by several factors. First, these studies measured hepcidin in children, whose degree of anaemia, which is defined by HB levels, is lower than adults in a majority of severe cases; second, cytokine levels in low-hepcidin severe malaria cases are lower than in uncomplicated cases, which suggests a lack of inflammation-driven stimulus for hepcidin production. In the present study, subjects with severe malaria had a robust pro-inflammatory response that may stimulate the expression of hepcidin.

Hepcidin is upregulated in response to several infectious and inflammatory conditions [39]. In a murine model, the production of hepcidin during a blood-stage infection can prevent a subsequent liver-stage infection; this inhibition was preserved in mice treated with anti-IL-6 antibodies [20]. Recently, it was shown that IL-10 and IL-6 are increased in primary macrophages co-cultured with *P. falciparum*-infected erythrocytes; IL-10 seemed to induce hepcidin in macrophages, which was mediated by signal transducer and activator of transcription 3-phosphorylation [40]. Also, malaria-infected red blood cells induced hepcidin mRNA synthesis by peripheral blood mononuclear cells, which indicates the importance of circulatory immune cells on hepcidin production [19]. Hepcidin was positively correlated with IL-6 and IL-10 in the mild malaria group in the present study. IL-10 is a regulatory cytokine and the correlation between hepcidin and this cytokine can modulate the clinical disease observed in mild malaria cases. Hepcidin was also associated with parasitaemia in subjects with mild malaria and hyperbilirubinaemia, which agrees with the results of other studies [21, 23]. The association between parasite burden and inflammatory response may explain the relationship between hepcidin and parasitaemia, although a direct effect of the malaria parasite on hepatic and/or macrophage hepcidin production cannot be ruled out [23]. Hepcidin was positively correlated with IFN-γ in subjects with severe malaria in the present study, which further suggests a role of immune response in hepcidin regulation. IFN-γ is a proinflammatory cytokine involved in malaria pathogenesis and symptomatology [33], and the correlation between hepcidin and this cytokine may influence malaria severity. Interactions between immunological factors and hepcidin may be different according to malaria outcomes; however, the small sample sizes in the severe malaria and malaria with hyperbilirubinaemia groups may have underestimated these associations.

This study was limited because of a small number of subjects in the severe and hyperbilirubinaemia groups, which may have underestimated the associations between the markers studied herein. Further, unfortunately, others iron parameters (i.e. ferritin, transferrin receptor) were not analysed to better understand the relation of hepcidin, immune response and iron metabolism.

## Conclusion

Hyperbilirubinaemia is a common clinical feature often associated with complications during malaria infection. The cytokine and chemokine responses in hyperbilirubinaemia are similar to the responses in uncomplicated malaria cases but less robust than the inflammatory responses observed in severe vivax malaria. Hepcidin levels are increased in cases of both severe malaria and malaria with hyperbilirrubinemia, and hepcidin levels are positively correlated with IL-6 and IL-10 in the mild malaria cases, and with IFN-γ in severe malaria subjects. These findings highlight the importance of immune response on hepcidin regulation. Hepcidin levels can be used to define iron supplementation programmes, but the findings from the present study suggest that hepcidin can also be used as a supplementary diagnostic marker of malaria infection.

## Additional files

10.1186/s12936-015-0930-x Distribution of cytokines and chemokines in mild malaria, hyperbilirubinaemia and severe malaria groups.
10.1186/s12936-015-0930-x Correlation parameters between the immune biomarkers according to study groups.
10.1186/s12936-015-0930-x Correlation parameters between the immune biomarkers and laboratory measures, hepcidin and parasitaemia in the study groups.

## Abbreviations

FMT-HVD: Fundação de Medicina Tropical Dr Heitor Vieira Dourado
PCR: polymerase chain reaction
RFLP: restriction fragment polymorphism
ELISA: enzyme-linked immunosorbent assay
HIV: human immunodeficiency virus
WHO: World Health Organization
IL-: Interleukin
IFN: interferon
TNF: tumour necrosis factor
CCL2: chemokine C–C motif ligand 2
CCL4: chemokine C–C motif ligand 4
GCSF: granulocyte colony stimulating factor
GMCSF: granulocyte macrophage colony stimulating factor
HB: haemoglobin
HT: haematocrit
PTL: platelets
AST: aspartate transaminase
ALT: alanine transaminase
OR: odds ratio
CI: confidence interval
HWE: Hardy-Weinberg equilibrium
AUC: area under the curve
ROC: receiver operating characteristic
SLC40A1: *ferroportin* gene
