# Supplementary material for: Associations between hepcidin and immune response in individuals with hyperbilirubinaemia and severe malaria due to Plasmodium vivax infection
Source: Malar J. 2015 Oct 14;14:407. doi: 10.1186/s12936-015-0930-x (PMC4607001; doi:10.1186/s12936-015-0930-x)
Supplement: Supplementary file 1 — 10.1186/s12936-015-0930-x Distribution of cytokines and chemokines in mild malaria, hyperbilirubinaemia and severe malaria groups. [file 12936_2015_930_MOESM1_ESM.docx]

**Supplemental Table 1. Distribution of cytokines and chemokines in mild malaria, hyperbilirubinemia, and severe malaria groups**

|  | **Mild malaria** | **Malaria with hyperbilirubinemia** | **Severe malaria** | **P-value** |
| --- | --- | --- | --- | --- |
| **Parameter (pg/mL) - median (IQR)** | **(n=52)** | **(n=14)** | **(n=17)** | **Kruskal-Wallis test** |
| **IL-1β** | 1.266 (1.266-1.266) | 1.266 (1.266-11.900) | 1.266 (1.266-88.040) | **0.0025** |
| **IL-2** | 0.461 (0.461-0.461) | 0.461 (0.461-48.180) | 48.600 (9.079-206.600) | **<0.0001** |
| **IL-4** | 1.407 (0.182-6.788) | 6.583 (1.101-21.460) | 6.788 (3.952-30.430) | **0.0034** |
| **IL-5** | 1.440 (1.440-1.440) | 1.440 (1.440-1.440) | 1.440 (1.440-11.190) | 0.3083 |
| **IL-6** | 1.037 (1.037-128.500) | 21.890 (1.037-73.750) | 58.860 (8.519-149.300) | 0.2457 |
| **IL-7** | 0.818 (0.818-28.350) | 0.818 (0.595-28.350) | 29.360 (0.818-112.700) | **0.0248** |
| **IL-8** | 8.602 (0.770-28.570) | 13.490 (6.118-28.610) | 16.180 (3.444-80.570) | 0.2510 |
| **IL-10** | 87.790 (14.190-468.600) | 53.440 (10.540-160.300) | 44.750 (8.004-124.400) | 0.2328 |
| **IL-12p70** | 1.390 (1.390-8.360) | 1.390 (1.390-221.100) | 221.100 (1.390-574.700) | **0.0005** |
| **IL-13** | 7.961 (1.213-79.610) | 20.460 (1.213-85.490) | 117.400 (69.150-204.000) | **0.0006** |
| **IL-17** | 1.222 (1.222-13.110) | 13.110 (1.222-49.230) | 73.390 (1.222-287.800) | **0.0158** |
| **IFN-γ** | 1.074 (1.074-97.880) | 1.074 (1.074-965.800) | 946.300 (1.074-1633.000) | **0.0304** |
| **TNF** | 22.980 (3.507-143.700) | 49.600 (31.950-413.600) | 197.500 (5.042-1081.000) | **0.0127** |
| **CCL2** | 141.700 (1.051-339.500) | 154.300 (58.570-187.800) | 167.000 (70.640-251.900) | 0.9326 |
| **CCL4** | 96.950 (47.380-471.200) | 103.700 (36.920-143.800) | 63.620 (22.010-178.100) | 0.1230 |
| **GCSF** | 74.310 (1.023-154.000) | 46.190 (6.900-196.700) | 74.310 (1.023-356.300) | 0.8265 |
| **GMCSF** | 0.514 (0.514-0.514) | 0.514 (0.514-0.514) | 0.514 (0.514-0.514) | 0.9557 |
| **Inflammatory ratios - median (IQR)** | |  |  |  |
| **IFN-γ/IL-10** | 0.076 (0.007-5.129) | 2.633 (0.014-23.120) | 8.234 (0.795-59.680) | **0.0177** |
| **TNF/IL-10** | 0.2471 (0.030-2.883) | 1.810 (0.336-11.530) | 4.942 (0.531-29.570) | **0.0024** |
| **(TNF + IFN-γ)/IL-10** | 0.750 (0.068-14.740) | 6.714 (0.735-147.000) | 14.930 (6.741-91.740) | **0.0033** |

IQR: interquartile range
